# Role of days postdelivery on breast milk production: a secondary analysis from the *EMPOWER* trial

**DOI:** 10.1186/s13006-019-0215-z

**Published:** 2019-06-04

**Authors:** Elizabeth V. Asztalos, Alex Kiss, Orlando P. daSilva, Marsha Campbell-Yeo, Shinya Ito, David Knoppert, EMPOWER Study Collaborative Group, Christoph Fusch, Lajos Kovacs, Annie Janvier, Georges Cauotte, Abhay Lodha, Barbara Bulleid, Doug McMilllan, Balpreet Singh

**Affiliations:** 1 Department of Newborn and Developmental Paediatrics, Sunnybrook Health Sciences Centre, University of Toronto, M4-230, 2075 Bayview Ave, Toronto, ON M4N 3M5 Canada; 2 Sunnybrook Research Institute, Sunnybrook Health Sciences Centre, University of Toronto, Toronto, ON Canada; 3 Perinatal and Women’s Health, London Health Sciences Centre, Western University, London, ON Canada; 4 School of Nursing, Departments of Pediatrics, Psychology and Neuroscience, Dalhousie University and Izaak Walton Killam Health Centre, Halifax, NS Canada; 5 Division of Clinical Pharmacology & Toxicology, Department of Paediatrics, Hospital for Sick Children, University of Toronto, Toronto, ON Canada; 6 School of Pharmacy (D.K.), University of Waterloo, Kitchener, ON Canada

**Keywords:** Breast milk production, Domperidone, Mothers of preterm infants, Galactogogue

## Abstract

**Background:**

With an increasing demand for mother’s own milk to be viewed as a primary source of nutritional support in the care of very small and preterm infants, mothers of preterm infants may be at risk of expressing suboptimal amounts of milk. The use of a galactogogue is often considered when these mothers are still having challenges in breast milk production.

**Methods:**

For this analysis, the study participants were the 90 mothers who participated in the *EMPOWER* trial and, at the time of randomization, were stratified by days post-delivery, 8–14 days and 15–21 days. The primary outcome measure was the proportion of mothers in each of the days post-delivery groups who achieved a 50% increase in breast milk volume on day 14 of the study treatment period.

**Results:**

There was no significant difference in the proportion of mothers in the 8–14 days group (75.0%) who achieved a 50% increase in breast milk volume on day 14 of the study treatment period.

compared to those in the 15–21 days group (60.9%), OR 1.93 (95% CI 0.78, 4.76; *p* = 0.15). Because comorbidities and exposure to antenatal corticosteroids between the groups of mothers were viewed as potential confounders, a logistic regression was performed after controlling for these two variables with the adjusted OR being 1.84 (0.73, 4.64; *p* = 0.19).

**Conclusions:**

This secondary analysis was able to demonstrate that mothers of very preterm infants, < 30 weeks gestation at birth, were able to respond to the study treatment in a similar fashion regardless of timing of entry and exposure to domperidone. In the presence of a suboptimal breast milk production by the end of the first week postpartum, below 250 ml/kg/d based on infant birth weight, a 14 day treatment of domperidone could be considered to augment breast milk production.

**Trial registration:**

*EMPOWER* has been registered at http://www.clinicaltrials.gov (identifier NCT 01512225) on January 10, 2012.

## Background

Over the past decade, there has been an increasing recognition of the role human milk plays in contributing to a reduction of major morbidities in the very small preterm infant [1–5]. This is especially true for necrotizing enterocolitis [6]. Although donor human milk has become more readily available for many infants, given the increased significant benefits associated with mother’s own milk, mothers are encouraged to provide their own pumped breast milk for their infants. These mothers may be at risk of expressing suboptimal amounts of milk for a variety of reasons [7–9]. Despite some known interventions to assist mothers of very preterm mothers to facilitate breast milk production [10], response to these approaches is varied and may not be adequate and the use of a galactogogue is often considered [11].

A recent trial, *EMPOWER*, was able to demonstrate that administration of a two week course of domperidone, initiated within the first 21 days after delivery, would lead to a modest increase in milk volume [12–14]. Although the results of the trial supported the use of a galactogogue, in this case domperidone, clarity regarding aspects of its use is still needed. Additional analyses from this trial have shown that response to domperidone is not affected by the pregnancy gestation at the time of giving birth [15]. Further analysis for this trial is underway to evaluate response patterns to domperidone with very specific volume groupings at trial entry (Asztalos EV, Kiss A, da Silva OP, Campbell-Yeo M, Ito S, Knoppert D: *EMPOWER* Study Collaborative Group. Early breast milk volumes and production: a secondary analysis from the *EMPOWER* Trial, submitted). For this secondary analysis, we sought to evaluate the role that timing of postdelivery initiation of domperidone played in a mother’s response to the treatment interventions in the trial.

## Methods

### Trial design

The *EMPOWER* trial has previously been described in detail [12, 13]. Briefly, the goal of *EMPOWER* was to reaffirm domperidone’s ability to increase breast milk volume to a clinically significant amount which required the use of a modified placebo arm. The trial was conducted in eight level III Neonatal Intensive Care units across Canada. The research ethics committee of each center approved the study protocol. All mothers who participated provided a written informed consent before being enrolled. Because the study was utilizing an off-label indication for domperidone, the study was conducted under the Food and Drug Act of Health Canada. The dose chosen for the trial was the dose (10 mg three times a day) supported by Health Canada and the most studied for the purpose as a galactogogue.

Mothers were eligible if their preterm infants were born ≤29 completed weeks gestation (23 ^0/7^–29 ^6/7^ weeks); were 8–21 days postdelivery; were pumping a minimum of six times a day in the four days prior to study entry; and, experiencing a milk volume that was < 250 mL/kg/d (based on their infant’s birthweight) during the previous 72 h period prior to study entry or a maternal report of milk volume reduction of 20% or more from a peak volume of the previous 72 h despite optimizing non-pharmacologic interventions. Centres utilized their own logs and volume documentation methods to determine eligibility into the trial which met Health Canada requirements.

All enrolled mothers were stratified by the number of days after giving birth, 8–14 and 15–21. Mothers were randomly assigned to one of two groups: Group A (domperidone 10 mg orally three times daily for 28 days); or Group B (placebo 10 mg orally three times daily for 14 days followed by domperidone 10 mg orally three times daily for 14 days). The primary outcome for the trial was based on the first 14 days of the study period for both groups.

Because of the trial’s pragmatic nature, the centers were encouraged to maintain their standard approach in supporting mothers related to the types of pumps utilized in each center. All centers had policies in place to encourage milk expression within a few hours after delivery and to help all mothers with breast milk provision during their infants’ neonatal hospitalization. During the study period, mothers were encouraged to pump 6–8 times in a given 24-h period; a diary specific for the trial was provided for the mothers to record their pumping times, volumes pumped, and any side effects they may experience. Non-pharmacologic (skin-to-skin, Kangaroo care, etc.) approaches were encouraged and followed standard procedures at each centre. Because of evolving concerns of cardiac Q-Tc interval prolongation related to domperidone at the time of trial initiation in 2011 [16], each mother had two electrocardiograms (ECGs) performed during the study period: at entry and at the end of the four week study medication period. The ECGs were reviewed centrally by a cardiologist, on prespecified time points during the study, to determine the presence of a significant Q-Tc interval prolongation during the week week study medication period.

### Study participants

For the purpose of this secondary study analysis, the study participants are the mothers as they were stratified in days post-delivery, 8–14 compared to 15–21 days, at the time of randomization.

### Outcome measures

The primary outcome for this secondary analysis was the proportion of mothers in each of the days postdelivery groupings who achieved the original primary outcome of the *EMPOWER* trial of a 50% increase in breast milk volume on day 14 of the study treatment period.

The secondary outcome measures for each group were: i) the proportion of mothers who achieved a 50% increase in breast milk volume on day 28 of the study treatment period; ii) the number of mothers who achieved a milk volume defined as 250 ml/kg/d on days 14 and 28 of the study treatment period; iii) the number of mothers who achieved an adequate milk volume defined as 500 ml/d on days 14 and 28 of the study treatment period; iv) the mean breast milk volumes on days 14 and 28 of the study treatment period; and v) the mean percent volume change from the start of the trial to day 14 and from day 15 to day 28 of the study treatment period.

### Analysis

The analysis was carried out using SAS Version 9.3 (SAS Institute, Cary, NC, USA) and followed an intention-to-treat analysis as in the original trial. Descriptive statistics were calculated for all variables of interest. Continuous measures were summarized using means and standard deviations, whereas categorical measures were summarized using counts and percentages. Chi-square and Fisher’s exact tests were used to compare categorical variables between groups. The primary and secondary outcomes were assessed between groups using a logistic regression model, controlling for comorbidities as listed in Table 1 and exposure to antenatal corticosteroids. Odds ratios were presented along with their associated 95% confidence intervals and *p* - values. Because milk volumes did not follow a normal distribution due to high variability, milk volume between groups was compared using the non-parametric Wilcoxon rank sum test.

**Table 1:** Baseline characteristics of mothers in the two postdelivery groups

Characteristic	8–14 days postdelivery *N* = 44	15–21 days postdelivery *N* = 46	*p* - value
Maternal age (years) (SD) (range)	32.1 (5.7)	30.7 (6.1)	0.29
	(19.7, 40.9)	(19.4, 44.6)	
Self-declared ethnicity, *n* (%)			
Caucasian	29 (65.9%)	30 (65.2%)	0.14
Black	2 (4.5%)	8 (17.4%)	
Asian	10 (22.7%)	6 (13.1%)	
Aboriginal	0 (0%)	1 (2.2%)	
Other	3 (6.8%)	1 (2.2%)	
Smoking prior to pregnancy, *n* (%)	16 (36.4%)	12 (26.7%)	0.33
Primigravida, *n* (%)	17 (38.6%)	14 (30.4%)	0.41
Comorbidities, *n* (%)	20 (45.0%)	26 (57.0%)	0.29
Hypertension Gestational/chronic)	4	8	
Diabetes (Gestational/Type I and II)	5	3	
Preterm Labour	5	7	
Chorioamnionitis	1	2	
Antepartum haemorrhage	7	3	
Other	9	9	
Antenatal corticosteroids, *n* (%)	40 (90.9%)	36 (78.3%)	0.09
Cesarean delivery, *n* (%)	24 (54.5%)	24 (52.2%)	0.82
Singleton Pregnancy, *n* (%)	37 (84.1%)	40 (86.9%)	0.77
Gestational age at delivery, mean (SD)	27.4 (1.7)	26.83 (1.6)	0.11
Infant weight at trial entry, mean (SD)	1016 (259)	906 (229)	0.03

Note: *p -* values were based on chi square test or Fisher exact test

*SD* Standard deviation

## Results

Between June 1, 2012 and June 30, 2015, 90 mothers were enrolled and equally allocated to the original trial study arms in *EMPOWER*. For this secondary analysis, there were 44 mothers in the 8–14 days post-delivery group and 46 in the 15–21 days group. The mean gestational age was 27.4 weeks and 26.8 weeks respectively.

Table 1 outlines the baseline characteristics of the mothers in the two groups of study. The two groups of mothers were similar in the characteristics except that the mothers in the 8–14 days group had a lower rate of comorbidities and the mothers in the 15–21 days group had a lower rate of exposure to antenatal corticosteroids, each not being statistically significant. The mean milk volume at the start of the study treatment was similar between the two groups of mothers, 100 ± 93 ml for the mothers in the 8–14 days group and 134 ± 91 ml for the 15–21 days group.

Table 2 shows the proportion of mothers in each group who achieved a 50% increase in expressed milk volume on day 14 of the study treatment. There was no significant difference in the proportion of mothers in the 8–14 days group (75.0%) compared to those in the 15–21 days group (60.9%), OR 1.93 (95% CI 0.78, 4.76; *p* = 0.15). Because comorbidities and exposure to antenatal corticosteroids between the groups of mothers were viewed as potential confounders, a logistic regression was performed after controlling for these two variables with the adjusted OR being 1.84 (0.73, 4.64; *p* = 0.19).

**Table 2:** Primary outcome: Proportion of mothers who achieved a 50% increase in milk volume on day 14 of study period in the two postdelivery groups

	8–14 days postdelivery *N* = 44	15–21 days postdelivery *N* = 46	Odds Ratio (95% Confidence Interval)	Adjusted Odds Ratio (95% Confidence Interval)^a^
Mothers who achieved a 50% increase in milk volume on day 14, *n* (%)	33 (75.0%)	28 (60.9%)	1.93 (0.78, 4.76), *p* = 0.15^*^	1.84 (0.73, 4.64), *p* = 0.19^*^

*p - values were based on logistic regressions

^a^adjusted odds ratio after controlling for comorbidities and antenatal corticosteroids

Table 3 shows the proportion of mothers in each group who achieved a 50% increase in expressed milk volume on day 28 of the study treatment. As in the primary outcome, there was no significant difference in the 8–14 days group (65.9%) compared to those in the 15–21 days group (65.2%), OR 1.03 (95% CI .43, 2.46; *p* = 0.95). There was no significant difference after adjusting for comorbidities and antenatal corticosteroid exposure.

**Table 3:** Secondary outcome: Proportion of mothers who achieved a 50% increase in milk volume on day 28 of study period in the two postdelivery groups

	8–14 days postdelivery *N* = 44	15–21 days postdelivery *N* = 46	Odds Ratio (95% Confidence Interval)	Adjusted Odds Ratio (95% Confidence Interval)^a^
Mothers who achieved 50% increase in milk volume on day 28, *n* (%)	29 (65.9%)	30 (65.2%)	1.03 (0.43, 2.46), *p* = 0.95^*^	0.92 (0.37, 2.28), *p* = 0.85^*^

*p - values were based on logistic regressions

^a^adjusted odds ratio after controlling for comorbidities and antenatal corticosteroids

Table 4 outlines the two additional measures of breast milk production evaluated: the proportion of mothers who achieved 250 ml/kg/d and an absolute value of 500 ml/d of breast milk at days 14 and 28 of the study period. For the achievement of 250 ml/kg/d on day 14 of the study period, there was no significant difference in the 8–14 days group (39.5%) compared to those in the 15–21 days group (45.2%), OR 0.79 (95% CI .33, 1.87; *p* = 0.60). There was no significant difference after adjusting for comorbidities and antenatal corticosteroid exposure. The proportion of mothers who achieved the target of 250 ml/kg/d on day 28 increased to 53.7% in the 8–14 days group and 52.5% in the 15–21 days group but remained clinically insignificant even after adjusting for comorbidities and antenatal corticosteroid exposure.

**Table 4:** Secondary outcome: Proportion of mothers who achieved 250 ml/kg/d and/or 500 ml/day on days 14 and 28 of study period in the two postdelivery groups

	8–14 days postdelivery *N* = 44 n (%)	15–21 days postdelivery *N* = 46 n (%)	Odds Ratio (95% Confidence Interval)*	Adjusted Odds Ratio (95% Confidence Interval)^a^
Number of mothers who achieved 250 ml/kg/d on day 14	17 (39.5%)	19 (45.2%)	0.79 (0.33, 1.87) *p* = 0.60	0.74 (0.30, 1.78) *p* = 0.50
Number of mothers who achieved 250 ml/kg/d on day 28	22 (53.7%)	21 (52.5%)	1.05 (0.44, 2.51) *p* = 0.92	0.97 (0.40, 2.34) *p* = 0.95
Mothers who achieved volume of 500 ml/day on day 14	4 (9.3%)	5 (11.9%)	0.76 (0.19, 3.05) *p* = 0.70	0.62 (0.15, 2.55), *p* = 0.51
Mothers who achieved volume of 500 ml/day on day 28	8 (19.5%)	10 (25.0%)	0.73 (0.25, 2.08), *p* = 0.55	0.68 (0.23, 1.99), *p* = 0.48

*p - values were based on logistic regressions

^a^adjusted odds ratio after controlling for comorbidities and antenatal corticosteroids

In evaluating the proportion of mothers who were able to achieve a volume of 500 ml/d, the proportion of mothers was low in both groups, 9.3% compared to 11.9% but doubled by 28 days reflecting the fact that, in this phase of the study treatment, all mothers were now allocated to receiving domperidone as part of the study protocol. Despite this, the numbers remained low with 19.5% in the 8–14 days group compared to 25.0% in the 15–21 days group.

Finally, Table 5 outlines the mean milk volumes achieved in both groups during the study period and the mean % volume change from the start of the study (day 0) to day 14 and also from day 15 to day 28 (end of the study treatment). Mean milk volumes on days 14 and 28 respectively were similar for both groups. Although the 8–14 days group demonstrated a higher per cent volume change from days 8–14, this was not found to be statistically significant.

**Table 5:** Secondary outcomes: Milk volumes at study time points days 14 and 28 in the two postdelivery groups

Milk volume parameters	8–14 days postdelivery *N* = 44	15–21 days postdelivery *N* = 46	*p -* value^*^
Milk volume at start of trial (ml) Mean (SD)	100.9 (80.9)	134.9 (105.0)	*p* = 0.16
Volume on study treatment day 14 (ml) Mean (SD)	237.1 (169.6)	251.1 (191.6)	*p* = 0.79
Volume on study treatment day 28 (ml) Mean (SD)	281.4 (202.3)	299.9 (237.0)	*p* = 0.85
Mean % volume change day 0 to day 14 (%) (SD)	256.8 (412.5)	173.1 (290.8)	*p* = 0.16
Mean % volume change day 15 to day 28 (%) (SD)	30.5 (42.4)	38.3 (73.7)	*p* = 0.90

* *p* - values based on Wilcoxon rank sum test

*Ml* Millilitres, *SD* Standard deviation

The total number of maternal adverse events recorded over the four week trial period was 54; there were no Q-T_c_ interval abnormalities noted on the maternal ECGs. Similarly, there were 28 outcome events recorded in the neonates, all of which were not related to the study intervention in the mother.

## Discussion

The *EMPOWER* trial, along with previous studies, reaffirmed the effectiveness of domperidone in increasing breast milk volumes and should be considered in mothers of preterm infants who are experiencing challenges with milk production despite adequate pumping support [12, 13, 17–20]. This secondary analysis shows that the response to domperidone is similar between the two timings of entry suggesting that the window of opportunity to achieve a response to domperidone can be achieved as early as the end of the first week postpartum but remains possible as far as 35 days postpartum. We look at several endpoints for this analysis, namely i) the number of mothers who experienced a 50% increase in breast milk volume at two time points (14 and 28 days); ii) the proportion of mothers who achieved a breast milk production of 500 ml/day; iii) achieving the trial entry volume of 250 ml/kg/d. These endpoints were chosen as they each may act as practical measures for clinicians to consider as they contemplate the use of a galactogogue with an individual mother or gauge an individual mother’s response if initiated on a galactogogue. Many NICU settings use a volume for nutritional support based anywhere between 120 and 160 ml/kg/d. If a mother is able to pump volumes of 250 ml/kg/d, she is pumping volumes that will be adequate for nutritional and growth support set at 160 ml/kg/d up to when her infant has reached close to 3.0 kg. We saw that regardless of timing entry into the trial, there were approximately 40–45% of the mothers who were able to achieve volumes of 250 ml/kg/d with the numbers rising to above 50%. A volume of 500 ml/day has often been cited as a goal to meet the nutritional needs of the preterm infant [21]. Regardless of when the mothers entered into the study, there was a significantly low number achieving this cited goal. This number did double by day 28 when we knew all the mothers were receiving domperidone as per the study protocol. Regardless of which measure is used to support mothers, it reinforces that these mothers remain at high risk of not being able to meet their infants’ nutritional needs fully and further support measures may need to be explored to help mothers achieve this goal. However, while further research is needed to determine ways to enhance mothers’ own milk in this vulnerable population, it is important to reiterate that any exposure to mother’s own milk has been associated with improved outcomes and as such the early use of domperidone may help to ensure at least some exposure to mother’s own milk [5].

We were able to show that the mothers in the ‘two time’ groupings responded similarly to the study treatment. Although the overall volumes achieved on days 14 and 28 did not differ between the two groups, there seemed to be a trend of improved volume as demonstrated by the % volume change in the earlier entry (8–14 days) group though this was not statistically significant. However, this may be clinically important as it may suggest that those mothers who may not be physiologically prepared for lactogenesis and are experiencing challenges very early on can respond adequately given this additional support. It may be prudent to evaluate mothers early in the postpartum period (8–14 days) and should their volumes fall below 250 ml/kg/d, based on their infants’ birthweight, to consider the use of a galactogogue, to help with milk production.

There are numerous maternal and obstetric factors which can modify how lactogenesis may occur in mothers of preterm infants [9]. Antenatal corticosteroids have been found to have an effect on breast milk volumes in particular in those women who have delivered at the very preterm gestational age [22]. We did not see an effect on how mothers responded to galactogogue support in this study with either a higher or lower exposure to antenatal corticosteroids. Similarly, differences in various comorbidities did not have an effect as have been reported [9].

There are strengths and limitations to this study. The findings of this study are based on a population of mothers who were recruited for the purposes of an already published randomized controlled trial [12–14]. The results of any ancillary study may be seen as having limited statistical power and prone to bias. A sample size of over 1200 mothers may be needed to detect an odds ratio of 0.75 with 80% power for some of the measures we attempted to evaluate. We were, however, reassured that our findings were not overly biased as our confidence intervals for our primary outcome were not overtly wide, the two groups of mothers in the analysis were similar in characteristics, and we did not see any effect from any potential confounding variables despite potentially being underpowered. Many factors can occur in the first 7–14 days post-delivery, particularly the timing of the initiation of pumping as well as the frequency of pumping not only in the first 3–4 days post-delivery but within the first 1–2 weeks, which could have an impact on breast milk production [23, 24]. Our goal for the trial and for this secondary analysis was to determine specific information to help clinicians determine whether a galactogogue was in order rather than be prescriptive on measures prior to determining consideration of a galactogogue.

## Conclusions

This secondary analysis was able to demonstrate that mothers of very preterm infants, < 30 weeks gestation at birth, were able to respond to the study treatment in a similar fashion regardless of timing of entry and exposure to domperidone. Lactation support, related in particular to pumping support, should be in place to assist these mothers to establish the autocrine function of the breast. Evaluation of these mothers during the first week postpartum should be in place to determine if volumes have remained low and, in particular below 250 ml/kg/d. If so, the addition of a galactogogue should be considered. A 14-day treatment of domperidone as early as eight days postpartum could be offered to augment breast milk production.

## Abbreviations

CI: Confidence interval
ml: milliliters
OR: Odds ratio
